# Cell Death-Associated Molecular-Pattern Molecules: Inflammatory Signaling and Control

**DOI:** 10.1155/2014/821043

**Published:** 2014-07-21

**Authors:** Beatriz Sangiuliano, Nancy Marcela Pérez, Dayson F. Moreira, José E. Belizário

**Affiliations:** Department of Pharmacology, Institute of Biomedical Sciences, University of São Paulo, 05508-900 São Paulo, SP, Brazil

## Abstract

Apoptosis, necroptosis, and pyroptosis are different cellular death programs characterized in organs and tissues as consequence of microbes infection, cell stress, injury, and chemotherapeutics exposure. Dying and death cells release a variety of self-proteins and bioactive chemicals originated from cytosol, nucleus, endoplasmic reticulum, and mitochondria. These endogenous factors are named cell death-associated molecular-pattern (CDAMP), damage-associated molecular-pattern (DAMP) molecules, and alarmins. Some of them cooperate or act as important initial or delayed inflammatory mediators upon binding to diverse membrane and cytosolic receptors coupled to signaling pathways for the activation of the inflammasome platforms and NF-*κ*B multiprotein complexes. Current studies show that the nonprotein thiols and thiol-regulating enzymes as well as highly diffusible prooxidant reactive oxygen and nitrogen species released together in extracellular inflammatory milieu play essential role in controlling pro- and anti-inflammatory activities of CDAMP/DAMP and alarmins. Here, we provide an overview of these emerging concepts and mechanisms of triggering and maintenance of tissue inflammation under massive death of cells.

## 1. Introduction

Apoptosis, necrosis, necroptosis, autophagy, and pyroptosis are different modalities of cell death programs that play important roles in regulation of the immune system [1]. Failure in cell death program leads to either increased numbers of lymphocytes, increased number of infected phagocytic cells, autoimmune diseases, or the inability to dampen immune response and malignancy. Different cellular stimuli, for example, TNF, Fas ligand, TRAIL ligand, double-stranded RNA (dsRNA), interferon-*γ* (IFN-*γ*), ATP depletion, ischemia-reperfusion injury, and pathogens have been shown to induce cell death and consequently the release of the of endogenous cell death-derived products [2–4]. These chemically distinct microbial and endogenous products are named as pathogen-associated molecular pattern (PAMPs), damage-associated molecular pattern (DAMPs), cell-death associated molecular patterns (CDAMPs), and alarmins [2–5]. Once released from dead and dying cells, they acquire adjuvant activity and cooperate with the cytokines: IL-1*α*, IL-*β*, IL- 33, TNF-*α*, TRAIL, IFNs, IL-8, and IL-12 and antibiotic peptides in augmenting innate response [2–5]. They are expressed in different immune and nonimmune cell types and are localized in the nucleus and cytoplasm, for example: high-mobility group box 1 (HMGB1), IL-1*α* (nucleus), S100 proteins, ATP, and uric acid (cytoplasm), heat shock proteins (exosomes), formyl peptides, mDNA (mitochondria) and heparan sulphate, and hyaluronan fragments (extracellular matrix). These mediators can act via different plasma membrane and intracellular recognition receptors and are part of host's normal protective response to tissue injury, sterile inflammation, and infection according to the “danger” model proposed by Polly Matzinger in 1994 [6].

It is still uncertain to what extent the many and overlapping functions of cytokines, microbial and damage- and cell death-associated molecular patterns, impact on the molecular switcher of the genetic program specifying Th17 and regulatory T cells (Tregs) and thereby impairing the Th1 or Th1 cell response. Overproduction of cytokines, PAMPs, DAMPs, and alarmins appears to restrict the growth of pathogens by fueling a potent immune response. However, in some situations, this response can be detrimental to the host and may contribute to autoimmune and autoinflammatory diseases and cancer [7]. Nonetheless, in some context, this could be a mechanism to prevent the initial proliferation of autoreactive cells, thus preventing autoimmune disease [8]. Along the acute and chronic inflammation is commonly observed cycles of proliferation and death of immune cells as well as the secretion of growth factors with survival and suppressor activity that exert regulatory effects on a relatively larger number of cell types in an autocrine and paracrine manner [7]. The proinflammatory mediator catabolism by the parenchymal/stromal cells reverts back the tissue to noninflammatory phenotype, which is called the resolution of inflammation [9]. Hence, resolution is an active rather than a passive process [9]. Therefore, futures studies to measure the impact of cell death subtypes and their products on the innate and adaptive response in the context of pathophysiological processes, such as acute and chronic inflammation, will be critical for designing new strategies to control inflammatory response in many diseases. Here, we will present an update on recent findings showing that various DAMPs/CDAMPs and alarmins act as direct mediators of the inflammation and have great impact on the outcome on the inflammatory response.

## 2. Inducers, Sensors, and Mediators of the Inflammatory Processes

The inflammatory reaction during the innate immune response is the first-line host-defense to pathogens such as bacteria, fungi, parasites, and virus [7]. The majority of pathogens can be detected by conserved and unique structural microbial components such as polysaccharides and polynucleotides that differ little from one pathogen to another but are not found in the host. The immune cells recognize such molecules referred to as PAMPs (or inducers) through one or more the pattern-recognition receptors (PRRs). These receptors (or sensors) contain a ligand-sensing region referred to as leucine-rich repeats (LRRs). The phagocytic leukocytes, endothelial and mucosal epithelial cells and antigen-presenting cells, and various targeted tissues express PRRs message and proteins during the inflammatory response. PRR families include the family of the Toll-like receptors (TLRs), the nucleotide-binding domain leucine-rich repeat-containing receptors (NLRs), C-type lectins (CTLs), RNA-sensing RIG-Like helicases (RLHs), and RAGE and DNA sensors [10–13]. The same repertoire of PRRs can recognize DAMPs originating from dying cells.

The PRRs share some molecular features and signaling pathways which are essential for their crosstalk and intracellular signaling that lead to activation of transcription of mediators and their receptors [12]. The mediators include inflammatory cytokines (TNF, IL-1*α*, IL-1*β*, and IL-6), chemokines (CCL2 and CXCL8), bioactive amines (histamine), lipid mediators from arachidonic acid (prostaglandins and leukotrienes), and products of proteolytic cascades, such as bradykinin and complement component 5a [7]. These mediators are in general short living molecules and act on target tissues, including local blood vessels, to induce vasodilation, extravasation of neutrophils, and leakage of plasma into the infected tissue. The acute inflammatory response is finished once the triggering insult is eliminated, the infection is cleared, and damaged tissue is repaired. Many PAMPs and DAMPs released in the inflammatory environment play activate roles in the subjacent processes that control and resolve the inflammation and promote repair and regeneration of tissues, promoting the reestablishment of the host homeostasis. The final resolution of inflammation is an active and highly regulated process orchestrated by specialized proresolving mediators derived from poly-unsaturated fatty acids [9, 14]. Next, we will present a short update on important aspects of PRRs family member and their signaling cascades.

### 2.1. TLRs

TLRs are expressed either on plasma membrane (TLR1, TLR2, TLR4, TLR5, and TLR6) or on endosomal membranes of endoplasmic reticulum (TLR3, TLR7, and TLR9) [12]. Each TLR specifically interacts with a rather diverse plethora of ligands, including bacterial flagellins, single strand (ss) RNA, double strand (ds) RNA, peptidoglycans, and imidazoquinoline compounds [12]. The intracellular adaptors and transducers involved in their signaling cascades have been described in detail elsewhere [5, 12]. After binding, the TLR intracellular TIR domain interacts with MyD88, TIRAP/Mal, or TRIF, which are proteins sharing a similar TIR domain. Upon stimulation, MyD88, via its death domain, interacts with the death domain of serine/threonine kinase (IRAK) family. IRAK1 and IRAK4 are activated by phosphorylation, leading to the dissociation of IRAK1 from the receptor complex. IRAK1 in turn interacts with the tumor necrosis factor receptor-associated factor (TRAF) family member TRAF6 and with TAK1 (transforming growth factor-*β*-activated kinase). The activation of TAK1 leads to the formation of the I*κ*B kinase and NF-*κ*B essential modulator complex (IKK/NEMO). The proteasome-mediated degradation of I*κ*B*α* is required for NF-*κ*B heterodimer (p50/RelA) activation and its translocation to the nucleus [11]. The NF-*κ*B then mediates the transcription of genes for cytokines such as IL-1*α* and *β*, IL-6, IL-8, TNF-*α*, IL-12, IL-15, IFN-*α* and IFN-*β*, cyclooxygenases (COX1 and COX2), inducible nitric oxide synthase (iNOS), chemokines, E-selectins, angiogenic factors, matrix metalloproteases, and genes for initiation of an adaptive immune response such as CD80, CD86, and CD40 [15]. The anti-cell death genes such as c-FLIP, c-IAP-1, c-IAP-2, A20, SOD2 (superoxide dismutase 2), and Bcl-X_L_ are also induced by NF-*κ*B. TLR3 and TLR4 and TLR7 and TLR9 also activate IRF3 (interferon regulatory factor 3) and IRF7, respectively, leading to the production of IFN-*α* and -*β* in a cell-type-specific manner [5, 12].

### 2.2. ILRs

The interleukin receptors IL-1R and IL-18R contain three extracellular immunoglobulin domains and one intracellular Toll/IL-1R homology (TIR) domain [16]. TIR domain interacts with MyD88, TIRAP/Mal, or TRIF. MyD88 interacts with the death domain of serine/threonine kinase (IRAK) family and, in turn, with NF-*κ*B essential modulator complex (IKK/NEMO), leading to NF-kB activation and production of inflammatory mediators [17].

### 2.3. NLRs

The nucleotide-binding domain and leucine-rich repeat-containing receptors (NLRs) consist of more than 23 members. The 14 neuronal apoptosis inhibitor proteins (NALPs or NLRPs), ICE protease activating factor (IPAF), NOD1 (or NLRC1) and NOD2 (or NLRC2) subfamily of cytoplasmic proteins share a central nucleotide-binding and oligomerization domain (NACHT) and differ in their N-terminal domains [10, 11, 18, 19]. NOD1 is constitutively expressed in a wide variety of cell types of both haematopoietic and non-haematopoietic origin, whereas NOD2 is predominantly expressed in monocytes, dentritic cells, Paneth cells, and intestinal epithelial cells [11]. They can recognize peptides derived from bacterial Gram-positive and -negative wall peptidoglycans, *γ*-D-glutamyl-meso-diaminopimelic acid (iE-DAP), and muramyl dipeptide (MDP). Both NOD1 and NOD2 transduce signals through the adapter protein receptor-interacting protein 2 kinase (RIPK-2), also known as RICK, which leads to NF-*κ*B downstream signaling and induction of proinflammatory cytokines. NOD2 is crucial to maintain microbial balance and its mutations increase the development of chronic inflammatory diseases [11].

NLRP1, 2, and 3 molecules harbor a central nucleotide-binding and oligomerization domain (NACHT), pyrin domain (PYD), acidic transactivating domain, or baculoviral inhibition of apoptosis protein repeat domain (BIR) and caspase recruitment domain (CARD) [10, 11, 16]. The PYD and CARD domains of the NLR/PYHIN receptors and the PYD and CARD domains of caspase-1 promote the interaction with an adapter molecular referred to as the apoptosis-associated speck-like protein (ASC). ASC forms a large protein aggregate, termed “ASC speck,” which provides a platform for the activation of caspase-1 [10, 11, 19]. Once activated, caspase-1 promotes the cleavage of the IL-1*β* precursor as well as the IL-18 precursor into active cytokines which are then released from secretory lysosomes or via cellular leakage. IL-1*β* binds to IL-1R and induces the same set of genes as do TLRs. Caspase-1 activation can help in tissue repair and release of many proteins without sequence signal such as IL-1*α*. Caspase-1 cleaves IL-33, but in this case, IL-33 is inactivated [16, 17].

NRLP3 can sense microbes and variety of endogenous “danger signals” such as uric acid crystals, basic calcium pyrophosphate dihydrate (BCP) crystals, hyaluronan, elevated extracellular glucose, and fibrillar amyloid-*β* peptide [16]. Moreover, various bacteria that secrete pore-forming toxins, such as* S. pneumoniae*, which produces pneumolysin, and* B. anthracis,* which produces anthrolysin O, as well as, the pore-forming channels such as nigericin, maitotoxin, and aerolysin are known to promote the cellular acidification and release of K^+^, thereby inducing the assembly of NALP3 inflammasome and caspase-1 activation [10, 11]. Extracellular ATP is a prototype NRLP3 inflammasome activator. It has been proposed that binding of ATP molecules to P2X7 purinergic receptor gated ion channel results in the recruitment and opening of a pannexin-1 membrane pore and intracellular K+ efflux, leading to activation of NRLP3 inflammasome [19].

Many NLRP3 activators increase the generation of ROS [20]. Thioredoxin-interacting protein (Txnip) interacts and promotes the inhibition of thioredoxins (Trx1 and 2), which are redox cytosolic (Trx1) and mitochondrial (Trx2) proteins capable of reducing thiols, thereby controlling damage induced by ROS [20]. After an increase in ROS, Txnip is released from oxidized Trx1 and in turn binds to NLRP3, which is essential for the NLRP3 inflammasome activation [21]. Notably, inflammasome activation is negatively regulated by mitophagy/autophagy confirming that inflammasome senses mitochondrial dysfunction [21].

### 2.4. CLRs

The C-type lectin-like receptor (CLR) is another family of transmembrane-associated innate immune recognition receptors. Examples of ligands for CLRs include the spliceosome-associated protein 130 (SAP130) and filamentous actin. CLR contains the C-type lectin-like domain (CTLD), a conserved structural motif arranged as two protein loops stabilized by two disulfide bridges at the base of each loop [12, 13]. The lectin activity of CLRs is mediated by conserved carbohydrate-recognition domains (CRDs), which contain four Ca^2+^ binding sites, and by an EPN (Glu-Pro-Asn) and QPD (Gln-Pro-Asp) motifs, which confer specificity for mannose- and galactose-based ligands, respectively [13]. CLRs are expressed preferentially by monocytes, macrophages, granulocytes, and dendritic cells. CLRs are distinguished from C-type lectin receptors in that CLRs have a major role in cell activation through their cytoplasmic signaling domain, or they acquire signaling features by association with other receptors, such as the TLR or Fc gamma receptor (FcR)-*γ* chain that has a receptor tyrosine-based activation motif (ITAM) within the cytoplasmic domain. CLRs family members include Dectin-1 (dendritic cell-associated C-type lectin-1), Dectin-2, macrophage-inducible C-type lectin (Mincle), the dendritic cell-specific ICAM3-grabbing nonintegrin (DC-SIGN), and DC NK lectin group receptor-1 (DNGR-1).

DNGR-1 is essential for MHC class I cross-presentation of dead-cell associated antigens, whereas Mincle recognizes SAP-130 [13]. Dectin-1 elicits a signaling cascade that begins with the tyrosine phosphorylation of the ITA, subsequently recruiting and activating the tyrosine kinase Syk. Syk induces production of ROS that act as microbicidal agents and contribute to the activation of the NALP3 inflammasome, leading to the production of IL-1*β*. Syk also recruits and activates CARD9/Bcl10 that in turn activates the canonical p65/p50 pathway. Dectin also activates the p38, ERK, and JNK cascades, as well as NFAT, all of which regulate gene transcription in cooperation with NF-*κ*B [13]. Dectin-1-Syk signaling induces DC maturation and secretion of cytokines, including IL-2, IL-10, IL-6, TNF-*α*, and IL-23, rendering DCs fully competent to direct priming of CD4+ T helper cells, CD8+ cytotoxic T cells, and antibody responses [13].

### 2.5. RIGs

The (RIG)-I-like receptors (RLRs) is comprised of retinoic acid inducible gene 1 (RIG-I), melanoma differentiation associated gene 1 (MDA5), and laboratory of genetics and physiology 2 (LGP2) member [13, 22]. These cytoplasmic CARD module-containing RNA helicase proteins function as intracellular sensors of RNA viruses. RLRs mediate type I and type III IFN expression through an adapter molecule, IFN-*β* promoter stimulator-1 (IPS-1), and subsequent activation of IRF3 and NF-*κ*B signal transduction pathways.

### 2.6. Cytosolic DNA Sensors

The recognition of cytosolic DNA appears to involve several sensors. The first identified cytosolic DNA sensor, named DNA-dependent activator of IFN-regulatory factors (DAI), binds cytosolic dsDNA and leads to the production of type I IFNs [22, 23]. DAI induces the production of type I IFNs through the TBK1/IRF3 pathway. The endoplasmic reticulum- (ER-) resident transmembrane protein stimulator of IFN genes (STING) functions as an essential signaling adaptor that coordinates the cytosolic detection of DNA to the TBK1-IRF3 signaling axis. STING is induced by an IFN-inducible ligase called TRIM56. The DNA sensor IFI16 recruits STING to activate a TBK1-IRF3-dependent pathway to IFN-*β* induction. STING is also known to recognize conserved products of microbial metabolism such as cyclic di-GMP, a universal bacterial second messenger released by microbial pathogens such as* Listeria monocytogenes*.

Absent in melanoma 2 (AIM2) is a member of the pyrin and HIN domain (PYHIN) family of cytosolic DNA receptor. AIM2 forms an inflammasome with ASC triggering caspase 1 activation and the subsequent of production of IL-1*β* and IL-18. DNA of various origins such as poly(dA : dT), plasmidic DNA and DNA from the bacterium* L. monocytogenes* have been shown to activate AIM2. Upon activation, AIM2 interacts with ASC, leading to the cleavage of caspase-1 and the secretion of IL-1*β* and IL-18 [22, 23].

It is becoming clear that these cytosolic DNA/RNA receptors play a major role in autoimmunity diseases more than in cell-death-induced acute inflammation [23].

### 2.7. RAGE

RAGE is a ~47–55 kDa protein, originally discovered as a receptor for advanced glycation end products (AGE). It is a multiligand receptor of the immunoglobulin superfamily that plays a key role in immune response and in the resolution of inflammation, tissue homeostasis, and repair/regeneration after acute injury [24–26]. It is expressed on monocytes, macrophages, T cells, DCs, smooth muscle cells, immature myofibers, endothelial cells, embryonic neurons and tumor cells. RAGE contains a single variable (V) domain containing two N glycosylation sites, followed by two constant (C1 and C2) domains, a transmembrane segment, and a short cytoplasmic tail necessary for ligand-induced signal transduction [24, 26]. RAGE needs to associate with adaptor proteins for intracellular signaling pathways which lead to the activation of NF-*κ*B, AP-1, CREB, STAT3, and NFAT transcription factors and thereby the inflammatory response and/or cell proliferation, survival, differentiation, and motility in a cell-specific manner [26].

**Figure 1:**
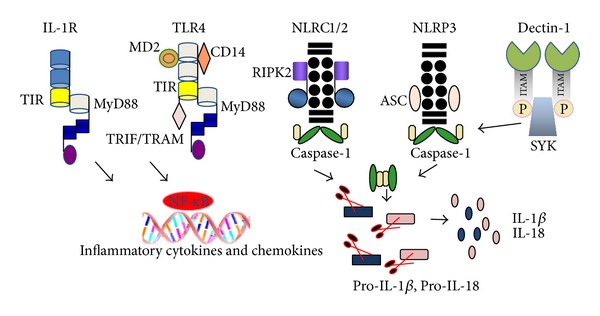
An integrative overview of intracellular signaling pathways by the IL-1R, TLRs, NLRs, and CLRs upon an innate immune response to microbial infection or cell death. Upon binding of PAMPs, DAMPs, or cytokines (IL-1*α* or IL-1*β*) the recruitment and assembly of high oligomeric platforms containing one more subunits of receptors, adaptors, inhibitors, ubiquitin ligase, and an initiator caspases (caspase-1, -8, or -5) occur. Following phosphorylation, ubiquitination, and degradation, certain components of each platform and an inflammatory signaling pathway are triggered. TLR and IL-1R receptor platforms recruit and activate MyD88 protein via intracellular Toll/IL-1R homology (TIR) domain. TIR domain interacts with MyD88, TIRAP/Mal, or TRIF/TRAM, which are proteins sharing a similar TIR domain. These proteins interact with the death domain of serine/threonine kinase (IRAK) family and IRAKs, which in turn promote the phosphorylation and activation the interferon regulatory factors (IRF1, 3, 5, 7), leading to the production of type I interferons (IFN-*α* and -*β*) in a cell-type-specific manner. IRAKs also cause the phosphorylation and activation of TAK1/TAB2/3 which in turn promote the phosphorylation of the inhibitor of NF-*κ*B (IKK subunits *α*, *β* and *γ*) and thereby NF-*κ*B activation. The different (NOD)-like receptors (NLRs), for example, NLRP3, interact with specific PAMPs or DAMPs to gather an inflammasome platform, via the ASC adaptor, which recruits and activates caspase-1, which in turn cleaves and releases IL-1*β* and IL-18. Some myeloid C-type lectin receptors (CLRs), for example, Dectin-1 recruits Syk through a phosphotyrosine in the hemITAM motif. Syk induces the activation of the NALP3 inflammasome, leading to the processing of pro-IL-1*β*. See text for further details.

RAGE acts as receptor to HMGB1 and S100 proteins and its interactions mediate NF-*κ*B-dependent production of the cytokines TNF*α*, IL-1*β*, and IL-6 and upregulation of the intercellular adhesion molecule 1 and the vascular cell adhesion molecule 1 on the surface of endothelial cells [27, 28]. On the other hand, new interest in RAGE comes from studies showing its ability to induce nervous system repair and cardiac muscle regeneration, which may depend on the local concentrations of its ligands [26].  Figure 1 shows an integrative overview of intracellular pathways by IL-1R, TLRs, NLRs and CLRs, as reviewed in this section.

## 3. Cell Death Types and Their Multiple Signaling Pathways and Immunological Consequences

The killing of cells is one of the most primitive host-defense techniques against intracellular infection. The cells dye by apoptosis, a physiological and regulated cell death process which is intrinsically tolerogenic (noninflammatory), or by necrosis, a pathological regulated cell death, which is inherently immunogenic and elicits an inflammatory reaction [1, 3, 29]. Pyroptosis, autophagy, and immunogenic cell death are other distinct processes recognized at morphological and biochemical levels. Genetic dissection in many organism and animal models provided us with knowledge of the early and late morphological and biochemical events of cell death programs. We will summarize our current understating of the multiple intracellular signal pathways and the consequences of subtypes of cell death in physiological and pathological processes.

### 3.1. Apoptosis

**Figure 2:**
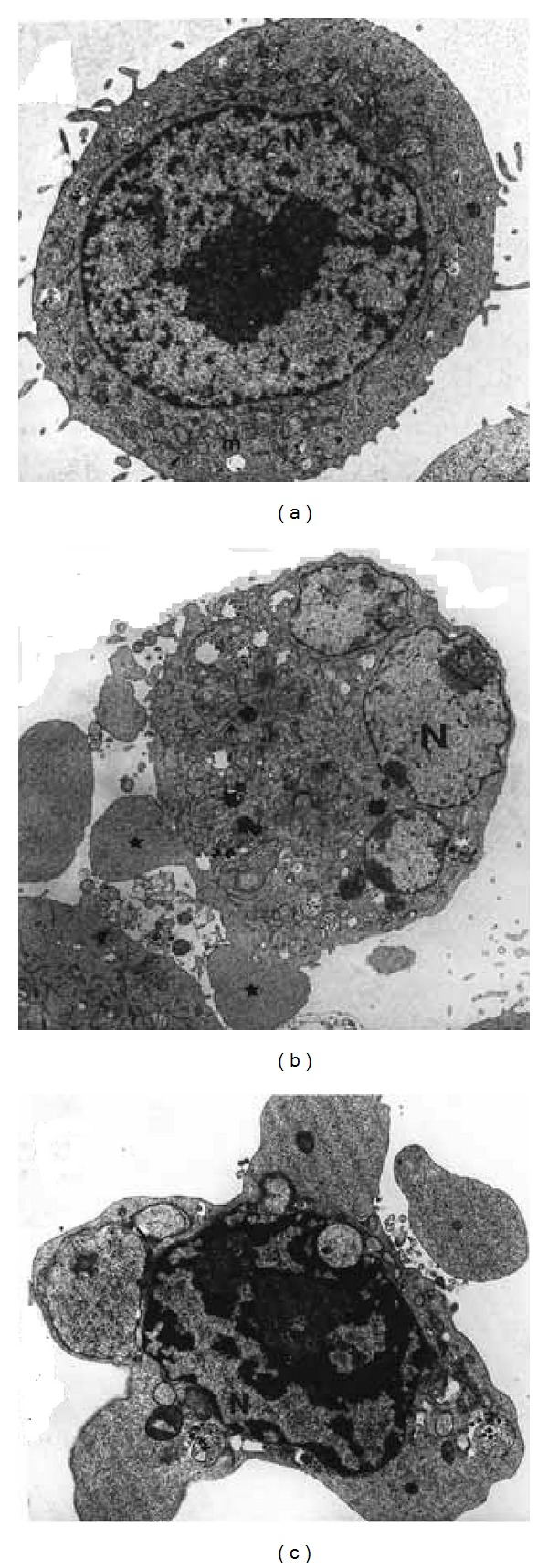
Ultrastructural changes associated with apoptotic cell death induced by TNF-*α* (10 ng/mL) in WEHI-164 cells, a murine fibrosarcoma cell line. Panel (a) shows a cell with intact cytoplasm and organelles. Panel (b) shows a cell in early apoptosis with extensive protrusion and blebbing of the plasma membrane, cytoplasmic shrinkage, and nuclear fragmentation. Panel (c) shows a cell with chromatin condensation and compact granular masses around the nuclear membrane, as well as multiple apoptotic bodies in the cytoplasm.

The complex cellular morphology known as apoptosis can be confidently recognized by a series of morphological changes at electron microscopy level. Apoptosis is characterized by cell shrinkage, membrane blebbing, condensation and margination of nuclear chromatin, degradation of DNA into nucleosomal units, and formation of apoptotic bodies (Figure 2). However, the hallmark of an apoptotic process is its dependence on caspase activation [1, 29].

Cells undergo apoptosis in response to extrinsic or intrinsic pathways which are regulated by various antiapoptotic and proapoptotic proteins [1, 30]. The extrinsic pathway is mediated by the tumor necrosis factor receptor (TNFR) superfamily. The interaction of the TNFR-1 with either FADD or pro-caspase-8 and -10, via both death domain (DD) and death effector domain (DED) triggers the apoptotic signaling cascade, whereas the interaction with negative regulator cFLIP (FADD-like IL-1*β*-converting enzyme-inhibitory protein) will block this apoptotic signaling cascade, leading to cellular survival and NF-*κ*B-mediated proinflammatory response (Figure 4).

The TNFR1 complex I comprises the adaptor protein TNFR1-associated death domain protein (TRADD), the death domain-containing protein kinase receptor-interacting protein 1 (RIPK1), and several ubiquitin E3 ligases, including TNFR-associated factor 2 (TRAF2) and cellular inhibitor of apoptosis protein 1 (cIAP1). The TNFR1 complex II comprises the adaptor FAS-associated death domain protein (FADD), caspase-8, and RIPK1. Activation of caspase-8 or -10 within either TNFR complex I or II propagates the activation of effector caspases-3, -6, and -7, which then cause cellular destruction without mitochondria participation (known as type I extrinsic pathway).

The intrinsic pathway is also called the mitochondrial pathway. Apoptotic cell death caused by mitochondrial dysfunction involves a rapid collapse of inner membrane potential, alterations of ions gradients due to loss or accumulation of metabolites and ions at different mitochondrial compartments, and the release of cytochrome c. These mitochondrial events seem to be either directly or indirectly regulated by the oligomerization and outer membrane permeabilization activity of BAX and BAK when BH3 ligands engage multiple Bcl-2-like relatives, thereby promoting their activation. Bax and Bak promote apoptosis by perturbing the permeability of the mitochondrial outer membrane (referred to as MOMP) and facilitating the release of cytochrome c, a cofactor for activation of caspase-9 that, in turn, activates the effector caspases-3, -6, and -7 [31]. In addition, several studies support the involvement of a putative mitochondrial permeability transition pore complex (PTPC) that regulates the inner membrane permeabilization in apoptosis [32]. The mitochondrial outer membrane ruptured could permeate the release of cytochrome c, but this remains controversial [33].

In vivo, apoptotic cells maintain their plasma membrane integrity and are rapidly phagocytosed in the absence of an inflammatory response. Apoptotic cells expose ecto-CRT, phosphatidylserine, HSP70, HSP90, opsonins, thrombospondin, HMGB1, and other molecules that serve to eat-me signals for professional APCs, monocytes and macrophages recognition, and engulfment [34]. The uptake of apoptotic cells by macrophages promotes cell growth and wound healing through the release of vascular endothelial growth factor (VEGF) and transforming growth factor-*β* (TGF-*β*), respectively [35]. This controlled process of apoptotic cell clearance is accompanied by suppressive effects on most immune cells under the effects of TGF-*β*1 and PGE_2_ [7].

### 3.2. Necrosis/Necroptosis

**Figure 3:**
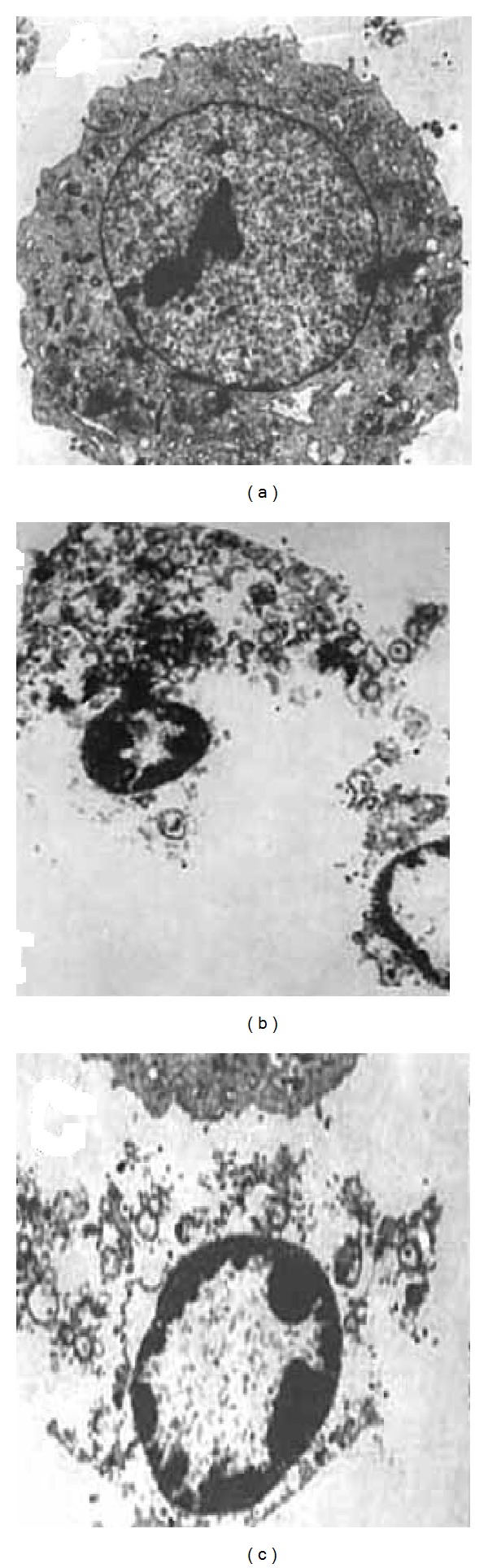
Ultrastructural changes associated with the necroptotic cell death induced by a combination of TNF-*α* (10 ng/mL) and z-VAD-fmk (20 *μ*M) in NHI3T3 murine fibroblasts (a diploid cell line). Panel (a) shows a cell with intact organelles and plasma membrane, whereas (b) shows cells with extensive cytoplasmic degeneration and plasma membrane rupture and (c) shows a cell with apoptosis-like chromatin condensation.

Necrotic death occurs quickly as a consequence of extreme physicochemical stress, such as heat, acidification, osmotic shock, mechanical stress, and freeze-thawing of cells [1]. Therefore, this cell death has been described as uncontrolled and accidental necrosis and is characterized by loss of plasma membrane integrity and cellular collapse (Figure 3). In necroptosis, there is no massive caspase activation. Necrotic cell death promotes inflammation and caspases exert a critical role as positive and negative regulators of inflammation induced by necroptosis [36].

**Figure 4:**
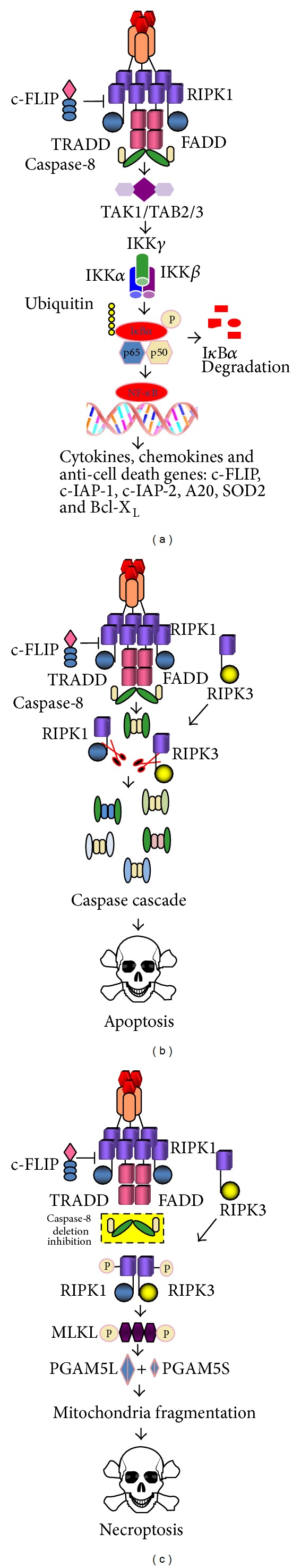
Schematic overview of TNF-*α*-induced signaling pathways to activation of NF-*κ*B (a), apoptosis (b), and necroptosis (c). TNF-*α* binding to TNFR causes the assembly of a membrane-proximal supramolecular complex including (but not limited to) TRADD, FADD, TRAF2/5 (TNFR associated factor 2/5), cIAP1/2 (cellular inhibitor of apoptosis 1/2), and RIPK1 (receptor interacting protein kinase). RIPK1 causes the phosphorylation and activation of TAK1/TAB2/3 which in turn promote the phosphorylation of the inhibitor of NF-*κ*B (IKK subunits *α*, *β*, and *γ*). The ubiquitination leads to the proteasome-mediated degradation of I*κ*B*α* and release and nuclear translocation of NF-*κ*B dimers. Recruitment and activation of caspase-8 play a crucial role in initiation of apoptotic (b) or necrotic cell death (c). Cleavage of both RIP1 and RIP3 by caspase-8 leads to apoptosis, whereas phosphorylation of RIP1 and RIP3 kinases causes the necrosome activation. Similarly, caspase-8 inhibition, or FADD/caspase-8 deletion, or RIPK3 induction leads to necrosome activation. Both RIPK1 and RIPK3 phosphorylate and activate a mixed lineage kinase domain-like (MLKL) and phosphoglycerate mutase family member 5 (PGAM5L and S) which recruit dynamin-related protein 1 (Drp-1), one of the key regulators of mitochondrial fission and mitochondrial fragmentation-dependent signaling pathway leading to necroptosis.

The inactivation of caspases proteolytic activity by Z-VAD-fmk, a pan-inhibitor Z-VAD-fmk, strongly sensitizes cell to TNF-*α*, TRAIL, and Fas-induced necroptosis [29, 37–39]. Necroptosis is inhibited by necrostatin-1, a small molecule inhibitor of RIPK1 (the receptor interacting protein kinase 1) that contains a death domain at the carboxyl terminus; thus, it is recruited to the TNF receptor 1 (TNFR-1). Smac mimetics are peptide antagonists of cIAP-1 (inhibitor of apoptosis), cIAP-2, and XIAP that can further enhance TNF-induced necrosis. The necrotic cell death occurs upon the assembly of a large, signal-induced multiprotein complex named ripoptosome that contains caspase-8, FADD, RIPK1, RIPK3, and MLKL (mixed lineage kinase domain-like, which then initiates the extrinsic necroptosis pathway [40]. The RHIM domain of RIPK1 and RIPK3 can form filamentous amyloid structures that are important for mediating necroptosis [41]. Caspase-8 inhibition blocks the cleavage of RIPK1 and RIPK3 allowing RIPK1 to phosphorylate RIPK3 and thereby the assembly of RIPK1-RIPK3 necrosome. This complex initiates the intrinsic necroptosis pathway with the participation of PGAM5L and PGAM5S (Figure 4). These two protein phosphatases cause the activation of dynamin-related protein 1 (Drp1) and its translocation to the mitochondria. In mammalian cells, mitochondrial fusion is regulated by mitofusin-1 and -2 (MFN-1/2) and optic atrophy 1 (OPA1), whereas mitochondrial fission is controlled by a dynamin-related protein 1 (Drp1). Along necrosis process, Drp1 associated with its mitochondrial anchors Fis1 (mitochondrial fission protein 1) and Mff (mitochondrial fission factor) to induce mitochondrial fragmentation; however, cytochrome c is not released as it occurs in the apoptosis intrinsic pathway [42]. Thus both mitochondrial fission and fusion proteins appear to modulate necroptosis through activities that are distinct from their roles in mitochondrial dynamics.

Activation of (RIPK1)-RIPK3 necrosome initiates the cascade of phosphorylation of several downstream target proteins including phospholipase A2, the proteases calpains and cathepsins, the cytoplasmatic NOXA1/NADPH oxidase complex, and the mitochondrial complex I, thereby leading to excessive ROS production, ATP depletion, and opening of the mitochondrial permeability transition pores [37]. These events are accompanied by prolonged JNK activation and RIPK3-induced stimulation of glycolysis, glycogenolysis, and glutaminolysis as well as the stimulation of Krebs cycle [38, 39, 43].

In the physiological condition, the FADD-caspase-8 platform prevents necroptosis. Mice deficient in FADD or caspase-8 die during embryogenesis; however, mice with triple deletion of FADD, caspase-8, and RIPK3 are viable [39, 40, 44, 45]. Therefore, FADD and caspase-8 act as prosurvival factors that suppress the deleterious effects of necrosis by promoting the cleavage and inactivation of RIPK1 and RIPK3. Programmed necrotic cell death occurs under various pathological processes such as ischemic brain injury, myocardial infarction, organ transplantation, and virus replication and is accompanied by strong inflammatory response. Studies on FADD-TNFR1 and FADD-MyD88 deficiency revealed that both TNF and TLR signaling partially contribute to progression of inflammation [39, 45, 46]. Studies on mouse models of the TNF-induced systemic inflammatory response syndrome (SIRS) and CLP-induced peritoneal sepsis have shown that multiple organ failure and animal mortality are driven by both RIPK1 and RIPK3-dependent necroptosis [29, 47].

Many human diseases are driven by activation of sterile inflammatory response, including ischemia-reperfusion injury, Alzheimer's disease, atherosclerosis, and toxic insults to liver and lung [2]. This response is accompanied by extensive necrosis of tissues. Necrotic cells release their cellular contents from organelles and nucleus (RNA, DNA, and nucleotides) as well as universal DAMPs such as IL-1*α*, HMGB1, ATP, uric acid, and HSPs that recruit and activate neutrophil, DCs, and macrophages, thereby promoting a highly inflammatory process [29, 34].

### 3.3. Immunogenic Cell Death

Immunogenic cell death (ICD) is a special type of cancer cell death elicited by some classes of anticancer chemotherapeutics, including oxaliplatin, mitoxantrone, bortezomib, and radiation and photodynamic therapy [48]. ICD promotes one inflammatory environment containing apoptotic and necrotic cells. The cell death-associated products released by these dying cells attract circulating dendritic cells (DCs) and other antigen-presenting cells (APCs). The uptake of dead cell-derived antigens by DCs and, consequently, the cytotoxic responses by effector T cells and NK cells contribute at least in part to success of therapy. Dead cells expose several proinflammatory signals including CRT on the plasma membrane and the release of ATP, HMGB1, HSP70, and HSP90 [48]. HMGB1 and ATP act in concert to promote IL-1*β* secretion by DCs. HSP70 and HSP90 enhance antigen cross-presentation and the release of proinflammatory cytokines. Large-scale clinical studies are been conducted to determine the prognostic value of ICD induced by current approved chemotherapy to different types of cancers [49, 50].

### 3.4. Pyroptosis

The activation of inflammasome containing intracellular engulfed bacterial and viral molecules induces pyroptosis which is a proinflammatory and cell death program dependent on caspase-1 activation [51, 52]. Pyroptosis is typically observed in infected macrophages, monocytes, and dendritic cells. It has been considered as a cell death modality with morphological and biochemical features of necrosis and apoptosis [51, 52]. The mechanism, characteristics, and outcome of caspase 1-dependent cell death are distinct from apoptosis. Along the pyroptotic program, a connected interplay of biochemical and morphological events causes the formation of pores (1-2 nm) in the plasma membrane, which leads to potassium efflux, water influx, cell swelling, and rupture of plasma membrane and release of intracellular contents [53].

It has been shown that during pyroptosis, caspase-1 and likely caspase-7 (21) act together in the proteolytic digestion of several types of proteins including chaperone HSP-90, *γ*-actin, ataxin-3, HnRNP-A2, and the glycolysis enzymes glyceraldehyde-3-phosphate dehydrogenase, enolase, pyruvate kinase, among others proteins [54]. Host cell death by pyroptosis contributes to the control of microbial infection such as* Salmonella*,* Shigella*,* Listeria*,* Pseudomonas*,* Francisella,* and* Legionella* [51, 52]. Pyroptosis is also caused by stroke and cancer therapy [53]. More studies are needed to identify which caspase-1 substrates have the ability to induce pyroptosis features in these pathologies.

### 3.5. Autophagy

Most cells infected by bacteria, such as* Shigella* and* Legionella*, viruses, and protozoa undergo autophagy. Autophagy is a cellular process involved in aberrant proteins and damaged organelles degradation by hydrolases into the lysosomes [55]. Autophagy plays a role in a wide variety of normal physiological processes including energy metabolism, organelle turnover, growth regulation, aging, and cellular self-digestion during starvation and hormone deprivation [55]. Along this process, organelles such as the mitochondria, endoplasmic reticulum, and protein aggregates are first enwrapped in double membrane vesicles, named autophagosomes, which deliver their content to endosomes and late to lysosomes. The formation of autophagic vacuoles is mediated up to 30 autophagy-related proteins codified by Atg genes, which were first identified in yeast [56]. The transition from diffuse cytosolic to punctuate pattern of the lipidated form of LC3 (Atg8) is used as one of the most reliable autophagy markers [55]. Notably, the membrane trafficking events required for autophagy also participate in pathogen delivery into the lysosome and into endosomal compartments containing the Toll-like receptors, such as TLR3, 7, 9, and 10. The formation of these complexes leads to activation of type I interferon signaling as well as delivery of endogenously synthesized viral antigens to MHC-II-processing and -loading compartments [56]. Therefore, autophagic cells are likely to instigate the biochemical events leading to innate and adaptive responses.

Autophagy has not been considered as modality of cell death; nonetheless, many stimuli that activate apoptosis induce autophagy, whereas signals that inhibit apoptosis inhibit autophagy [56]. The pan caspase inhibitor Z-VAD-fmk inhibits caspases but also blocks lysosomal cathepsins and hence cell death by autophagy. Antiapoptotic proteins, such as Bcl-2 family members, bind to and inhibit beclin (Atg 6), and proapoptotic factors, such as BH3-only proteins, disrupt this inhibitory interaction and thereby activate autophagy or vise-versa [56]. Autophagy is triggered by ROS derived from either the mitochondrial electron transport chain or NAPDH oxidases. Autophagy of damaged mitochondria limits ROS-modulated caspase-1 activation and seems to negatively regulate pyroptosis [53]. Mitophagy is a specialized form of autophagy in which mitochondria are specifically targeted for degradation at the autophagolysosome [57].

It is becoming increasingly clear that endoplasmic reticulum- (ER-) stress induces autophagy (Boland et al., 2013). Calcium depletion, oxidative damage, and energy depletion cause ER stress, leading to the unfold protein response (UPR) via three major transmembrane proteins: pancreatic ER kinase- (PKR-) like ER kinase (PERK), activating transcription factor-6 (ATF6), and inositol-requiring enzyme 1 (IRE1). These sensors are sequencing-activated after their dissociation from chaperone GRP78 [58]. ATF6 controls the synthesis of the genes encoding ER-associated protein degradation (ERAD), whereas PERK suppresses protein synthesis by phosphorylating eukaryotic initiation factor 2*α* (eIF2*α*). Activated IRE1 activates the transcription factor X-box binding protein 1 (XBP-1). Together, these transcription factors orchestrate the UPR. If severe enough, each of these stimuli can result in cell death; IRE1 facilitates apoptosis by recruiting ASK1 and JNK (c-Jun N-terminal kinase). Therefore, there are many lines of evidence connecting UPR, cell death and magnitude, and duration of inflammatory process.

## 4. Thiol Oxidation Modulates the Immunological Activities of CDAMPs/DAMPs and Alarmins

Reactive oxygen species (ROS) and nitrogen species (RNS) regulate a wide variety of signaling pathways including anti-inflammatory responses and adaptation to hypoxia [59]. ROS/RNS can cause damage to all biomolecules (proteins, lipids, and DNA) and ultimately lead to cell death [60]. Reactive oxygen species are produced mainly by two sources: transmembrane NADPH oxidase (NOX family) and the mitochondrial electron transport chain (ETC) macromolecular complexes [59]. Oxidation of sulfur alters chemical reactivity and metal binding properties of proteins, and it can serve as a molecular switch to control protein structure and function [60]. Redox-active cysteine residues in protein are subject to more than one kind of modification, which include disulfide, glutathionyl, nitrosyl, or sulfenic acid modification [61, 62]. S-glutathionylation is the formation of disulfide between the cysteine of glutathione and the cysteine moiety of a protein, also known as protein-mixed disulfide or PSSG [61].

**Figure 5:**
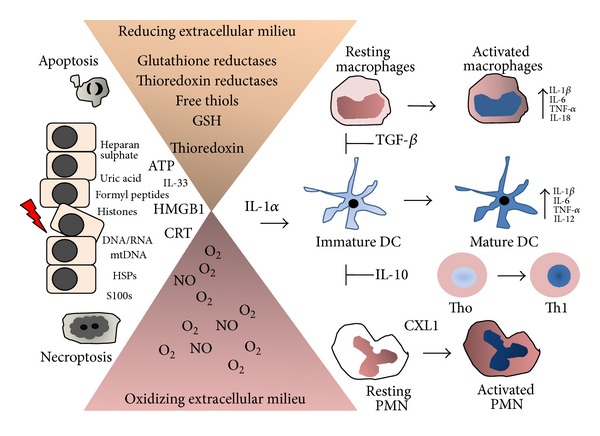
Inflammatory response to the cell death-associated factors release by apoptotic and necrotic cells and their control by the redox inflammatory microenvironment. Dying and dead cells release a wide array of molecules, including ATP, CTR, HMGB1, nuclear DNA, and IL-1*α* that exert multiple functions in the innate immune response. IL-1*α* is most potent danger factor. These cells also release nonprotein thiols and thiol-regulating enzymes and highly diffusible prooxidant reactive oxygen and nitrogen species, which create an oxidizing and reducing gradient in the in extracellular milieu. The pro- and anti-inflammatory activity of DAMPs, alarmins, cytokines, and lipid mediators are affected by oxidation/reduction reactions in certain components of their structures. For instance, if all cysteines in the HMGB1 protein are reduced, it binds to CXCR4 and acts as chemoattractant. HMGB1 linked with disulfide binds to TLR-4 and induces proinflammatory cytokines, whereas further cysteine oxidation to sulfonates by ROS abrogates both activities. The constitutively higher ROS levels in macrophages were shown to inhibit procaspase-1 activation by reversible oxidation of cysteine residues on the zymogen. Similarly, IL-1*β* and IL-18 are prone of oxidation, causing their inactivation. Thus, the outcome of inflammatory response depends on the balance of oxidizing and reducing factors confined within injured cells that are released in the extracellular milieu. Inflammation resolution occurs with efficient macrophage clearance of the apoptotic cells. Apoptotic cells emit a small number of DAMPs, alarmins, and cytokines as well as proteases, as compared with apoptotic cells. Thus, necrotic cells sustain a long inflammatory response throughout the process of cellular resolution. Futures studies focusing on molecular targets involved in the critical steps of necrotic cell death program may help us to find new therapeutics to control the deleterious inflammatory response.

The cytotoxic potential of ROS is controlled by various mitochondrial, cytosolic, and peroxisomal antioxidant systems [60]. There are two different intracellular redox compartments into cells, the endoplasmic reticulum and peroxisomes/mitochondria that consisted of highly oxidizing organelles, as opposite, the cytoplasm and nucleus which are very decreasing compartments due to the presence of the thioredoxin peroxidase-thioredoxin reductase and glutathione peroxidase-glutathione reductase systems. The normal extracellular environment is highly oxidizing, and following types of injuries such as ischemia, O_2_ deprivation and infarction, the release oxidoreductases and thiols affect the composition and properties of extracellular environment and this may sustain and prolong the inflammatory reaction [63]. The critical role of the redox state of the injured tissue in the regulation of cytokines and various cell death-associated molecules has been previously proposed [63]. A simplified model is present in Figure 5.

Specific antibodies and chemical are available for detection S-glutathionylation (Cys-SSG), S-nitrosylation (Cys-SNO), sulfonated cysteine (Cys-SO_3_^−^), and sulfenic acid modified proteins. These tools have been used in proteomic and immunochemical methods for monitor global changes in cysteine oxidation of proteins in diverse normal and pathological conditions [61]. One study has identified the transition cysteine to sulfenic acid in over 175 proteins bearing cysteine modification [61, 64]. In the list there are important cell death-associated molecules with known role in the innate and adaptive immune system, including chaperones belonging to HSP60, 70, 75, and 90 kDa families, calnexin (CNX), and calreticulin (CRT). The functional consequences of oxidative cysteine modifications of important CDAMPs/DAMPs and alarmins in the context of inflammation are described below.

### 4.1. HMGB1

HMGB1 is a chromatin component of 27 kDa that is structurally composed of three different domains: two homologous DNA-binding sequences entitled box A and box B and a highly, negatively charged C terminus. It is involved in V(D)J recombination by acting as a cofactor of the RAG 1 and 2 complex [65, 66]. HMGB1 lacks a classic signal sequence, although there is homology to a nuclear localization sequence that may participate in the nuclear functions of the protein. HMGB1 was discovered as a late mediator of endotoxin lethality [65]. Administration of HMGB1 to mice mediates the development of fever, anorexia, and sickness behavior [65].

Acting as cytokine, HMGB1 transduces signals and coordinates cellular activities through (RAGE), TLR2, TLR4, TIM-3, chemokine CXC receptor (CXCR)-4, and CD24-Siglec G/10 [66]. Addition of HMGB1 to monocyte and macrophages stimulates the synthesis of TNF-*α*, IL-1*α*, IL-1*β*, IL-1ra, IL-6, IL-8, macrophage-inflammatory protein-1*α* (MIP-1*α*), and MIP-1*β* but not the synthesis of IL-10 or IL-12. HMGB1 can interact with ssDNA, LPS, IL-1*β*, and nucleosomes and amplifies TLR-mediated inflammatory responses possibly by binding to TLR2, TLR4, TLR9, IL-1R, and RAGE. HMGB1 can induce DC maturation as evidenced by increased CD83, CD54, CD80, CD40, CD58, and MHC class II expression [48, 66]. HMGB1 is also a proliferative signal for both human CD4+ and CD8+ T-cells. Confirming these important roles of HMGB1,* Hmgb1*-deficient mice die at as early as E15 day as result of hypoglycaemia, reduced autophagy, and lack of DC-induced inflammatory response, critically important for survival in the neonatal period [65, 66].

HMGB1 has a role in the pathogenesis of a variety of sterile inflammatory conditions including rheumatoid arthritis, lupus erythematosus, and Sjögren syndrome, trauma and hemorrhagic shock, and ischemia-reperfusion injury of the liver, heart, kidney, and brain [65, 66]. HMGB1 adopts different redox states under oxidative stress and the oxidation serves as a feedback mechanism to control the proinflammatory activity of HMGB1 in vivo [67]. HMGB1 contains three conserved cysteines, which are sensitive to oxidation: Cys23, Cys45, and Cys106. For instance, if all cysteines in the HMGB1 protein are reduced, it binds to CXCR4 and acts as chemoattractant. HMGB1 linked with disulfide binds to TLR-4 and induces proinflammatory cytokines, whereas further cysteine oxidation to sulfonates by ROS abrogates both activities [68]. On the other hand, reduced HMGB1 binds to RAGE, induces Beclin-1-dependent autophagy, and promotes resistance to chemotherapeutic agents or ionizing radiation, while oxidized HMGB1 increases the cytotoxicity of certain chemotherapeutics via induction of apoptosis [50]. Furthermore, various reports have indicated that HMGB1 is involved in tumor tissue invasion and metastasis by recruiting macrophages and endothelial cell precursors [48].

### 4.2. HSPs

Within cells, HSPs act as chaperone and protect proteins against acute denaturation and aggregation, which could cause proteotoxicity [62]. Exposed to membranes, ecto-HSP70 and HSP90 are important inducers of the immunogenicity of stressed and dying cells. Extracellular HSP70, HSP90, and gp96 are peptide carriers, inducers of cytokines, and stimulants for immune cells during stress. These proteins, for instance, have been described to bind to TLR4 and to CD14, which are lipopolysaccharide membrane protein receptors. HSPs induce the maturation of dendritic cells and present peptide molecules to antigen presenting cells (APCs), thus linking the innate immune and adaptive immune systems. On the other hand, HSP peptides can be a route to induction of regulatory T cells (Treg) which inhibit reactive T cells, as well as, IL-10, thereby contributing to anti-inflammatory response [69]. HSP70 has anti-inflammatory properties including downregulating inflammatory cytokine production, increasing cell and tissue tolerance of cytokine-induced cytotoxicity [69].

One report has shown that the binding of peptides is more pronounced for HSP70 than for Hsc70 and is accompanied with a gradual change in secondary structure under oxidative conditions [70]. HSP70 and HSP90 contain cysteine residues in which oxidation induces change to a conformation with high chaperone activity [64]. It is not known what happens to these HSPs immune functions once owing this modification if, for example, this leads to an anti-inflammatory or proinflammatory phenotype.

### 4.3. CRT

CRT is a 46-kDa Ca^2+^ binding ER-resident proteins that participate in Ca^2+^ storage in the lumen of the ER [71, 72]. The role of CRT in Ca^2+^ homeostasis is critical since* crt*-deficient mice die at E14.5 embryonic age. Interesting,* crt*-deficient mice are rescued by the constitute expression of calcineurin [72]. CRT, gp96 (grp94 or endoplasmin), protein disulfide isomerases (PDIs), immunoglobulin-heavy-chain-binding protein (BiP/GRP78), and GPR78 are also ER folding factors (or chaperones), aiding newly synthesized ER proteins in proper folding. Some folding factors, such as ERp57 and PDI, are oxidoreductases that catalyze proper disulfide bond formation in protein substrates to aid in their folding [73]. CRT possesses a lectin domain capable of binding to misfolded proteins and glycoproteins. CRT and CNX (calnexin) interact with the ERp57 to promote disulfide bond isomerization in bound unfolded glycoproteins. CRT contains a highly redox-active cysteine that undergoes sulfenic acid modification [64]. Therefore, oxidized calreticulin itself might drive protein folding in ER by promoting disulfide formation [64].

Under apoptotic stress, CRT associates with phosphatidylserine (PS) in a Ca^2+^ dependent manner and both are translocated as punctate clusters on the cell surface. The CRT/PS complex is recognized by a variety of receptors and adaptor molecules and function as most notable eat-me signal of apoptotic cells [34, 48]. CRT binds to CD91/LRP1 to trigger macrophages to secrete cytokines, stimulates dendritic cells to express antigen presenting costimulatory molecules, and is involved in wound healing [71]. The mechanism by which CRT is released in extracellular space is only speculative; change in pH and/or calcium levels may be involved in the release of CRT from the ER. CRT is sensitive to sulfenic acid modification and it can affect both its function and its subcellular location [64], a hypothesis that deserves to be evaluated.

### 4.4. S100 Proteins

There are over 24 homologous intracellular S100 proteins, which are characterized by calcium-binding EF hand motifs, low molecular weights, ability to form homodimers, heterodimers and oligomers, and tissue-specific expression [74]. Binding of Ca^2+^ and binding of Zn^2+^ are known to induce major conformational changes in S100 proteins, leading to an exposure of hydrophobic patches that interact with protein targets involved mainly in the cytoskeleton and cell proliferation. S100 proteins can bind to G-protein-coupled receptors, scavenger receptors, or heparan sulfate proteoglycans and N-glycans [74]. S100A7 (psoriasin) is overexpressed in inflammatory skin diseases and is induced in keratinocytes by IL-17 and IL-22 and flagellin via TLR7 [74]. S100A8 and S100A9 are present in neutrophils, monocytes, and myeloid progenitors and can be induced in keratinocytes during inflammation. The S100A8 and S100A9 form a heterocomplex (known as calprotectin) that bind to carboxylated glycans and RAGE, leading to intracellular NF-*κ*B activation and thereby the expression of cytokines that act as growth factors for tissue repair and regeneration [75, 76]. S100A8/A9 and S100A12 induce prothrombotic and proinflammatory responses in endothelial cells including induction of thrombospondin, chemokines, and adhesion molecules [77]. Human S100A8/S100A9 is chemotactic for neutrophils and influences migration of other cell types, including myeloid-derived suppressor. S100A8/S100A9 induces proinflammatory cytokine TNF-*α*, IL-1*β*, IL-6, and IL-8 in macrophages via NF-*κ*B activation [78]. S100A8/A9 and S100A12 are elevated early in tissues and serum immune pathological conditions associated with inflammation such as arthritis, inflammatory bowel disease, vasculitis, multiple sclerosis, psoriasis, and cystic fibrosis and are considered suitable biomarkers of inflammation [78]. S100B-RAGE is one important cellular signaling to severe pulmonary infection by* Aspergillus fumigatus* [79]. The sustained activation of the S100B-RAGE signaling is also involved in the hypoxia-induced inflammation in cystic fibrosis [80].

It has been reported that S-nitrosylation and S-glutathionylation regulate the activity of some representative S100 proteins. One study indicated that S100A1 and S100B activities are controlled by nitrosylation at Cys-84 [81]. Another study showed that S-glutathionylation of S100A1 at the single Cys-85 leads to a 10-fold increase in the affinity of the N- and C-loops of the protein for Ca^2+^ binding [82]. Human S100A8 is chemotactic for neutrophils and the activity may depend on its oxidation state [76]. S-nitrosylated S100A8 reduces mast cell activation and mast cell-mediated leukocyte adhesion and transmigration in the microcirculation in vivo [76]. S100A8/S100A9 can induce pro- and anti-inflammatory actions which are linked to their oxidation states [75, 76]). The S-glutathionylated S100A9 suppresses neutrophil migration [75].

### 4.5. IL-1*α* and IL-1*β*

The IL-1 family (IL-1F) comprises 11 members and the most studied are IL-1*α* and IL-1*β* [17]. IL-1*β* is a potent pyrogen that induce leukocyte tissue migration and expression multiple cytokines and chemokines. Interleukin-1 receptor antagonist (IL-1Ra) is a specific inhibitor of IL-1*α* and IL-1*β*. The gene deletion studies showed that mice deficient for IL-1*α* and IL-1*β* as well as for caspase-1, IL-6, and TNF- *α* and TNFR1 are viable and do not develop spontaneous diseases. In contrast, mice deficient in IL-1Ra develop arthritis. Thus, these cytokines are needed for infections, trauma, and immunological reactions [17]. Interleukin-1*α* is the most power danger signal released upon necrosis that exerts effects on both innate and adaptive immunity [2, 83]. IL-1*α* is a chromatin-associate cytokine and is highly dynamic in the nucleus of living cells. During apoptosis, intracellular IL-1*α* concentrates in dense nuclear foci and is not released along with cytoplasmic contents. IL-1*α* precursor is a signal peptideless protein; it is not readily secreted and only released from cells undergoing necrosis [17]. IL-1 binds to 1 IL-1 receptor (IL-1R1) leading to multiple proinflammatory effects including cytokine secretion, neutrophil recruitment, and upregulation of major histocompatibility complex (MHC) and costimulatory molecules on antigen presenting cells [17].

The influx of neutrophils towards necrotic dendritic cell-derived products from a sterile inflammation (in the absence of pathogens) is mediated through IL-1*α* [2]. The necrosis induced IL-1*α* inflammatory activity is highly cell type dependent [83]. The IL-1*α* precursor is released by hypoxic cells and incites an inflammatory response by recruiting myeloid cells into the area [17]. IL-1*α* stimulates the production of chemokines CXCL1 and CXCL2, which are involved in the neutrophil recruitment. The neutrophil migration along the inflammation to sterile necrotic cell death depends on both IL-1*α* and IL-1*β*. CD11b+ macrophages are required to produce IL-1*α* and bone-marrow-derived cells are required to produce IL-1*β* [83]. IL-1*β* is processed by caspase-1 in NLRP3 inflammasome and also leukocytes serine proteases and calpains [17]. IL-33, a IL-1 family member, is secreted by necrotic cells independent of caspase-1 and caspase-8 or calpain, but it is inactivated by caspase-1 [17].

It is interesting that IL-1*α*, IL-33, and HMGB-1 act as DNA-binding cytokines allowing the access of several transcription factors, including steroid hormone receptors, p53/p73 complexes and recombinases for genomic DNA repair and modification [83]. Therefore, it seems like that these cytokines act as DNA damage sensor during stress and prevent nuclear breakdown and release of inflammatory DNA and histones from nucleosomes; to date only limited information is available about these mechanisms.

## 5. Conclusions and Future Directions

This short review has highlighted the intricately and connected extrinsic and intrinsic molecular pathways and alternative ways to induce cell death programs named apoptosis, necrosis/necroptosis, and pyroptosis. Autophagy can modulate these programs by limited control of damage organelles such as mitochondria.

In vivo studies, in particular, in response to a variety of stress conditions and chemical insults, have furthered the knowledge and acceptance that cell death plays a multifaceted modulatory role in both innate and adaptive immune responses. The immunogenic cell death has emerged as cancer cell death modality stimulated by certain chemotherapeutic regime that exerts antineoplastic effects by eliciting a novel or reestablishing a preexisting antitumor immune response. The sterile necrotic cell death is a cell death modality in the absence of pathogen that elicits an innate immune-mediated acute inflammation.

Dying and death cells release in extracellular milieu an increasing number of nuclear-, cytosol-, and mitochondria molecules that stimulate an inflammatory response. Among them, HMGB1, CRT, HSPs, and IL-1*α* are the most relevant to date. These cell death-associated molecules trigger their inflammatory signaling pathways by binding to TLRs, NLRs, CTLs, RLHs, and other membrane and intracellular receptors present in immune and nonimmune cells. These leads to activation of the inflammasomes and NF-*κ*B transcription factor, which in turn promote the synthesis and release of proinflammatory cytokines and other mediators connecting cell death and magnitude and duration of inflammatory reactions. Caspases act as anti-inflammatory enzymes serving to limit the potentially proinflammatory consequences of cell death. A major goal in future studies is the development of specific antagonists able to dismantle assembly of these signaling platforms.

Overproduction and release of the self-molecules by stressed, dying, and dead cells influence the development and outcome of diverse range of diseases, including atherosclerosis, inflammatory bowel disease, diabetes, obesity, and inflammatory neurodegenerative disorders. Similarly, the overproduction and release nonprotein thiols and thiol-regulating enzymatic systems as well as reactive oxygen and nitrogen species exert important positive and negative control in diseases-associated inflammation in part by modulating the cell death-associated factor immunogenic activities. Even though antioxidants do not hold much potential to treat inflammatory diseases, many new small peptides and compounds for control cell death are under development and might yield clinical benefits.

Although there is much information about the diverse, sometimes pleiotropic effects of mediators of inflammation, future studies will define the interplay and cooperative role of cytokines, chemokines, lipid messengers, and cell death-derived mediators in cell survival, inflammation, or cell death. Elucidating the critical nodes in the cell death signaling pathways will help us to design and develop new therapeutic strategies for acute inflammation and inflammatory disorders.
